# Citric Acid in the Passivation of Titanium Dental Implants: Corrosion Resistance and Bactericide Behavior

**DOI:** 10.3390/ma15020545

**Published:** 2022-01-12

**Authors:** Pablo Verdeguer, Javier Gil, Miquel Punset, José María Manero, José Nart, Javi Vilarrasa, Elisa Ruperez

**Affiliations:** 1 Bioengineering Institute of Technology, International University of Catalonia, Josep Trueta s/n, 08195 Barcelona, Spain; pverdeguerm@gmail.com; 2 School of Dentistry, Universitat Internacional de Catalunya (UIC), C/Josep Trueta s/n, Sant Cugat del Vallès, 08125 Barcelona, Spain; josenart@uic.es (J.N.); jvilarrasa@uic.es (J.V.); 3 Biomaterials, Biomechanics and Tissue Engineering Group (BBT), Department of Materials Science and Engineering, Universitat Politècnica de Catalunya (UPC), Av. Eduard Maristany 16, 08019 Barcelona, Spain; miquel.punset@upc.edu (M.P.); jose.maria.manero@upc.edu (J.M.M.); elisa.ruperez@upc.edu (E.R.); 4 Barcelona Research Centre in Multiscale Science and Engineering, Technical University of Catalonia (UPC), Av. Eduard Maristany, 10-14, 08019 Barcelona, Spain; 5 UPC Innovation and Technology Center (CIT-UPC), Technical University of Catalonia (UPC), C/Jordi Girona 3-1, 08034 Barcelona, Spain; 6 Institut de Recerca San Joan de Déu, Hospital Sant Joan de Deu (IRSJD), 08034 Barcelona, Spain

**Keywords:** citric acid, dental implant, passivation, corrosioan, bacteria, periimplantitis, wettability, contact angle (CA), surface free energy (SFE)

## Abstract

The passivation of titanium dental implants is performed in order to clean the surface and obtain a thin layer of protective oxide (TiO_2_) on the surface of the material in order to improve its behavior against corrosion and prevent the release of ions into the physiological environment. The most common chemical agent for the passivation process is hydrochloric acid (HCl), and in this work we intend to determine the capacity of citric acid as a passivating and bactericidal agent. Discs of commercially pure titanium (c.p.Ti) grade 4 were used with different treatments: control (Ctr), passivated by HCl, passivated by citric acid at 20% at different immersion times (20, 30, and 40 min) and a higher concentration of citric acid (40%) for 20 min. Physical-chemical characterization of all of the treated surfaces has been carried out by scanning electronic microscopy (SEM), confocal microscopy, and the ‘Sessile Drop’ technique in order to obtain information about different parameters (topography, elemental composition, roughness, wettability, and surface energy) that are relevant to understand the biological response of the material. In order to evaluate the corrosion behavior of the different treatments under physiological conditions, open circuit potential and potentiodynamic tests have been carried out. Additionally, ion release tests were realized by means of ICP-MS. The antibacterial behavior has been evaluated by performing bacterial adhesion tests, in which two strains have been used: *Pseudomonas aeruginosa* (Gram–) and *Streptococcus sanguinis* (Gram+). After the adhesion test, a bacterial viability study has been carried out (‘Life and Death’) and the number of colony-forming units has been calculated with SEM images. The results obtained show that the passivation with citric acid improves the hydrophilic character, corrosion resistance, and presents a bactericide character in comparison with the HCl treatment. The increasing of citric acid concentration improves the bactericide effect but decreases the corrosion resistance parameters. Ion release levels at high citric acid concentrations increase very significantly. The effect of the immersion times studied do not present an effect on the properties.

## 1. Introduction

Dental implants are designed to achieve primary mechanical stability as a result of mechanical interlock of bona and implants, as well as to promote a strong bone to implant interaction over time through osseointegration [1,2,3]. Thus, the long-term success of dental implants largely depends on rapid healing with safe integration into the jaw bone [4]. Albrektsson et al. suggested six key-factors that are crucial for the success establishment of reliable osseointegration: surface conditions, implant material and design, status of the bone, surgical technique, and implant loading conditions [5]. In the last few decades, many researchers have made significant efforts in order to increase the success rate of dental implants, focusing their efforts on the control of surface properties in order to both stimulate osseointegration and decrease healing times [6,7].

Thereafter, a large number of scientific research works have been carried out in order to assess the influence of implant surface properties on bone healing. As a result of the studies described above, several factors of great importance related to both osseointegration and bound healing have been identified. The aforementioned key-factor list of surface properties includes surface chemistry, morphology, topography, wettability, surface energy and charge, crystal structure, roughness, chemical composition, strain hardening, residual stress, thickness of titanium oxide layer, as well as the presence of impurities, metal and non-metal composites and coatings [8]. The characterization of these parameters and their improvement will be the key to the success of the titanium dental implant [9,10,11,12,13,14,15,16]. Among these, wettability and free surface energy of an implant surface are considered to be very crucial.

Assuming that the surface properties are the key-factors influencing long-term success of dental implants, biocompatibility, speed and quality of osseointegration as well as wound-healing period, can be modulated through their modification [7,17]. As a result, a wide range of surface modification techniques have been developed, optimized and finally applied to commercially available dental implants during the last decades [3,17], which have been summarized in several reviews [3,4,7,18,19,20,21]. The development of the dental implant sector has been evolving in a parallel way to the development and successful implementation of the different above mentioned surface modification techniques, which has been recently classified by Hanawa et al., in five different generations [22]. In summary, surface modification processes have evolved over time from initial first-generation mechanical processes (turning and grinding), continuing towards morphological-based second-generation processes (grooving, sandblasting, chemical acid etching, laser abrasion and anodic oxidation), moving towards the development of third-generation physicochemical active surfaces (HA-coatings and chemical treatments), and finally evolving to the development of both fourth-generation biochemical active surfaces (Collagen, peptides and BMP immobilization) and fifth-generation biological active surfaces (stem cells and tissues coatings) [7,22].

Dental implants are placed most probably in the highest aggressive biological human medium, within are exposed to a complex biological and electrolyte environment, as well as to extremely high mechanical loading forces due to mastication or even bruxism [23]. Biological and electrolyte oral cavity environment are affected by a wide range of factors including bacteria oral microbiota and dental plaque, saliva, gastric acids, as well as by changing levels of oxygen, temperature and pH. [24,25]. These harsh service conditions promote the action of a wide range of degradation mechanisms including corrosion, ion-release and wear of dental implant materials than can cause undesired toxic and allergic related side effects, which can compromise the durability or lifespan of dental implants [26]. In addition to the foregoing, oral cavity shows probably the largest human microbiome with more than 700 microbial species described [27,28], which can produce dental oral diseases such as periodontitis and tooth decay that may lead to teeth loss [29,30,31,32,33]. Despite the high success rate of titanium dental implants even higher than 95% at 10 years of implantation [34], lack of osseointegration and bacterial infection can lead to device failure [35,36,37,38,39]. Consequently, there is a strong need to develop new strategies to combat biofilm-related implant infections in order to improve the long-term implant success rate [40,41,42], without necessarily resorting to the use of systemic antibiotic prophylaxis to prevent antibiotic resistant bacteria (ARB) related problems [43,44,45].

Some previous research has pointed to the importance of surface energy and cleanliness in the initial stages of tissue-healing after implantation, when the presence of inadequate levels of surface energy and contaminants (impurities) may compromise speed and quality of osseointegration [3,46,47,48,49,50,51]. In conjunction with the above considerations, a careful control of implants surface chemical composition has been progressively increasing its relevance in order to produce high-quality devices. As a consequence of such above-mentioned research, an initiative of manufacturers and researchers was launched recently [52].

The use of citric acid in oral implantology is often related to disinfection effect for periodontal diseases due to its good antibacterial properties. Some studies on the use of citric acid as an antimicrobial agent due to its efficacy against biofilms formed on titanium can give some indications of the effect of citric acid on the surface of titanium [53,54]. The immersion of Ti in citric acid can lead to a slight increase in roughness. This increase in roughness does not lead to an increase in bacterial recolonization as the roughness remains below 0.2 μm, a value below which bacterial adhesion is not affected [55]. Citric acid is characterized by its high concentration and low pH, yet it does not alter cellular activity on the Titanium surface. It is used as a disinfectant as it is able to remove biofilms without causing damage to periodontal tissues [56]. Htet et al. [56] demonstrated the bactericide character of citric acid using laser treatment, reflecting the great potential of citric acid treatment for disinfection of the anodized implant surface.

Passivation is, in general, an oxidation reaction obtained by chemical or electrochemical process which promotes the formation and increasing of the thickness of protective layers [14,15,16,57]. This treatment serves to increase the thickness of the oxide layer, increasing the corrosion resistance of the galvanic couples with the metal of the abutment as well as to exert an integral cleaning on the titanium surface. Some researchers have pointed out that the oxidation process changes the characteristics of the TiO_2_ oxide layer transforming it into a more biocompatible [21]. The effect of passivation and oxidative agents and the role of titanium oxide as the physico-chemical characteristics of the surface are poorly studied and understood. Several chemical agents, electrochemical process, laser treatments have been tested [56,57,58,59] but there is no consensus in relation to the chemotherapeutic agent to optimize the cleaning, corrosion resistance and at the same time to produce a decreasing of ion release and the inhibition of the bacteria adhesion.

The main aim of this contribution focuses on the evaluation of the effect of the acid passivation treatment on both surface properties and antibacterial capacities of “Commercially pure” Ti-cp grade 4 samples, comparing two different acids (conventional hydrochloric and newly citric acid treatments) with a non-treated control group. In addition to the primary objective, the secondary aim of this research is related to determine the effect of both concentration and immersion time parameters on citric acid passivation. All of the study groups of samples were thoroughly characterized in terms of roughness, wettability, surface energy, corrosion resistance and ion release behavior. Moreover, biological response was evaluated by means of bacterial viability adhesion assays using two different bacterial reference strains, Pseudomonas aeruginosa (gram-) and Streptococcus sanguinis (gram+), to evaluate the feasibility for its application to titanium dental implants.

## 2. Materials and Methods

### 2.1. Materials

One hundred and twenty flat disc samples of commercially pure Ti (cp) of grade 4 (KLEIN, Bienne, Switzerland) were provided by the company SOADCO S.L (SOADCO, Escaldes Engordany, Andorra) have been used.

The six sample groups were defined as follows:

- Control. As-received material.
- HCl: The discs were immersed in a solution of hydrochloric acid (HCl) 20% (v) for 40 s at room temperature (HCl group). This type of passivation is the very common in the implants and prosthesis.
- Citric acid 20% 10′. The discs were immersed in a solution of citric acid 20% (v) for 10 min at room temperature.
- Citric acid 20% 20′. The discs were immersed in a solution of citric acid 20% (v) for 10 min at room temperature.
- Citric acid 20% 30′. The discs were immersed in a solution of citric acid 20% (v) for 10 min at room temperature.
- Citric acid 40% 10′. The discs were immersed in a solution of citric acid 40% (v) for 10 min at room temperature.

After treatment, a total of three sequenced ultrasonic cleanings (3 min) were carried out: two with distilled water and one with ethanol.

### 2.2. Methods

#### 2.2.1. Confocal Laser Scanning Microscopy (CLSM)

Roughness evaluation of all study groups of samples were analyzed by means of non-contact and non-destructive three-dimensional confocal laser scanning microscopy using an Olympus LEXT OLS3100 (OLYMPUS Corp., Shinjuku-ku, Tokyo, Japan) confocal microscope. Three different samples (*n* = 3) of each group of study (*n* = 6) were analyzed by means of three measurements per sample at ×1000 magnification. The parameters Ra (arithmetic average height) and Rz (average value of the absolute values) were determined. Ra corresponds to the arithmetic average mean of the absolute values of the deviations of the profiles of a given length of the sample. Rz corresponds to the sum of the maximum peak height and the maximum valley depth within the sampling length. [60].

#### 2.2.2. Contact Angle and Surface Free Energy

Wettability and surface energy of samples were measured using a Contact Angle System OCA15plus (Dataphysics Instrument Company, Filderstadt, Germany) and results were analysed with SCA20 software (Dataphysics Instrument Company, Filderstadt, Germany) [11,61,62]. Contact angle (CA) and surface free energy (SFE) were determined by using the traditional Sessile Drop measurement method in the static mode. The aforementioned process allows the measurement of the angle θ formed between the water drop and the surface. The greater the contact angle, the lower the wettability and vice versa. For angles less than 10° the surface is considered superhydrophilic, for angles between 10° and 90° hydrophilic and for angles greater than 90° hydrophobic. A droplet generation system equipped with a 500 μL Hamilton syringe with micrometric displacement control was used to control the volume (3 μL) and to deposit the droplet.

Two different reference liquids were used to calculate the surface energy, measuring the contact angle values using ultra-distilled Milie-Q grade (Millipore Milie-Q Merck Millipore Corp., Darmstadt, Germany) as a polar liquid and di-iodomethane (Sigma Aldrich, St. Louis, MO, USA) as a non-polar liquid, respectively. The contact angle measurements of di-iodomethane have been obtained following the same procedure as for water [63].

The surface energy was calculated using (Equation (1)) the Owens and Wendt equation [11,61,62,64,65]:

(1) $$\gamma_{L}\cdot (1+\cos\theta )=2\cdot ({\left(\gamma_{L}^{d}\cdot \gamma_{S}^{d}\right)}^{1/2}+{\left(\gamma_{L}^{p}\cdot \gamma_{S}^{p}\right)}^{1/2})$$

where *γ_d_* and *γ_p_* represent the dispersive and polar components respectively of the liquid used and is the angle between the solid and the liquid. The total surface energy of a surface equals the sum of its dispersive and polar components.

#### 2.2.3. Electrochemical Measurements

**Figure 1:**
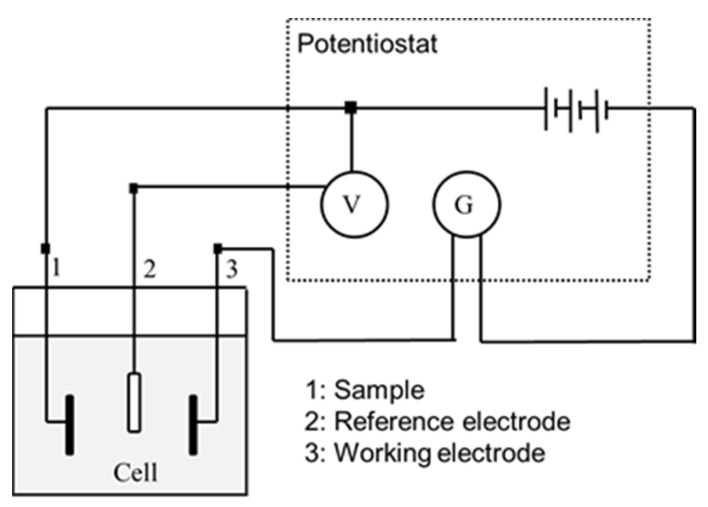
Experimental set up used for corrosion resistance.

Corrosion behavior of samples was evaluated by means of electrochemical measurements, conducting open circuit potential (OCP) measurements as well as by Cyclic potentiodynamic polarization curves determination. The electrochemical cell used was a polypropylene (PP) container with a capacity of 185 mL and a methacrylate lid with six holes for the introduction of the sample, the reference electrode and the counter electrode. For both the open circuit potential measurement tests and the potentiodynamic tests, the reference electrode used was a saturated calomel electrode (SCE), with a potential of 0.241 V compared to the standard hydrogen electrode. All tests were performed at room temperature and in a Faraday cage to avoid the interaction of external electric fields. The experimental setup can be seen schematically in Figure 1.

For the open-circuit potential (OCP) measurement tests, only the sample and the reference electrode were placed in the electrochemical cell. Tests were carried out for 5 h for all of the samples, taking measurements every 10 s during the whole test procedure. The potential was considered to be stabilized when the variation of the potential is less than 2mV over a period of 30 min according to ASTM G31 standard [66]. With this test, it was determined which samples are more noble (higher potential) and which are more susceptible to corrode. The data and the E-t curves were obtained using the PowerSuite software with the PowerCorr-Open circuit test mode.

Cyclic potentiodynamic polarization curves were obtained for the seven study groups following the ASTM G5 standard specifications. In this test, a variable electrical potential is imposed by the potentiostat between the sample and the reference electrode, causing a current to flow between the sample and the counter electrode. The counter electrode used was platinum [67,68].

Before starting the test, the system was allowed to stabilize by means of an open-circuit test for 1 h. After stabilization, the potentiodynamic test was launched, performing a cyclic sweep from −0.8 mV to 1.7 mV at a speed of 2 mV/s. These parameters were entered into the PowerSuite program using the PowerCorr-Cyclic Polarization function to obtain the curves. The parameters studied were:

- icorr (μA/cm^2^)/corrosion current density.
- Ecorr (mV)/Corrosion potential: value at which the current density changes from cathodic to anodic.
- Erep (mV)/Repassivation potential: potential at which the passive layer regenerates.
- Ep (mV)/Pitting potential: value at which pitting corrosion may occur.
- ip (μA/cm^2^)/passivation current density.
- irep (μA/cm^2^)/repassivation current density.

The results were plotted in the Evan’s diagram (LogI-E) in order to properly determining Ecorr and icorr parameters by extrapolating the Tafel slopes. These slopes also allow us to obtain the Tafel coefficients: anodic (*βa*) and cathodic (*βc*). These coefficients represent the slopes of the anodic and cathodic branch respectively. In accordance with the ASTM G102-89 standard [69], obtaining these values allows us to calculate the polarization resistance (*Rp*) using the Stern-Geary expression (Equation (2)) and the corrosion rate (CR in mm/year) using (Equation (3)), respectively [70,71].

(2) $$Rp=\frac{\beta a\cdot \beta c}{2,303\cdot (\beta a+\beta c)\cdot i_{corr}}$$

The polarization resistance indicates the resistance of the sample to corrosion when subjected to small variations in potential. A total of 30 potentiodynamic tests were carried out, obtaining at least 5 curves per group.

(3) $$CR=K_{1}\cdot \frac{i_{corr}}{\rho }\cdot EW$$

Ten different samples (*n* = 10) of each group of study (*n* = 6) were used for corrosion behavior evaluation. The test area was 19.6 mm^2^. The electrolyte used for all of the tests was Hank’s solution (Sigma Aldrich, St. Loius, MO, USA) which is a saline fluid that artificially reproduces the ion composition of the human physiological environment. Its composition is shown in Table 1.

#### 2.2.4. Ion Release

Ion-release behavior was evaluated according to ISO 10993-12 standard, quantifying Ti-ion released by means of inductively coupled plasma-mass spectroscopy (ICP-MS) using a Perkin Elmer Optima 320RL equipment (Waltham, MA, USA). Five samples (*n* = 5) from each study group (*n* = 6) have been used to ion-release tests.

After weighing the samples (m = 0.206 g) a weight adjustment was made at the rate of 1 mL of Hank’s solution for each 0.20 g of sample, according to ISO 10993-5 standard [69]. The 5 samples of each group were placed in the same Eppendorf with 5 mL of Hank’s solution and stored at 37 °C. Sample incubation was carried out using an incubator oven MEMMERT BE500 (MEMMERT Gmbh, Scheabach, Germany). Hank’s solution (Sigma Aldrich, St. Loius, MO, USA) extracted and stored in the refrigerator after 1, 3, 7, 14, and 21 days.

After each extraction, 5 mL of fresh Hank’s solution has been replenished into the Eppendorf containing the samples. All Eppendorfs were used after a thorough cleaned be cleaned with 2% Nitric Acid and dried before use. Ti elemental calibration standards were prepared by serial dilution containing Ti-ions at least five different concentrations from 1 ppb to 1 ppm using elemental stock solutions (NIST).

#### 2.2.5. Bacterial Strains and Culture Conditions

Bacterial assays were carried out with two different oral pathogens representing a Gram-negative and a Gram-positive bacterial strain, respectively. *Pseudomonas aeruginosa* was used as a Gram-negative bacterial strain model and was obtained from Colección española de cultivos tipo (CECT 110, Valencia, Spain). *Streptococcus sanguinis* was used as a Gram-positive bacterial strain model and was obtained from Culture Collection University of Gothenburg (CCUG 15915, Goteborg, Sweden).

A total of six samples (*n* = 6) have been used for the bacterial adhesion test for each study group of samples, three samples from each study group were used for the Gram-positive and three for the Gram-negative.

The culture media and material (PBS) were previously sterilized by autoclaving at 121 °C for 30 min using autoclave oven SELECTA model Sterilmax (SELECTA, Abrera, Spain). Prior to the adhesion test, the samples were also sterilized. For this purpose, three 5-min washes were carried out in sterile culture plates. After removing the ethanol, the samples were exposed to ultraviolet light for another 30 min [72,73].

The agar plates were cultured at 37 °C for 24 h. From this culture, the liquid inoculum was prepared by suspending the bacteria in 5 mL of BHI (Brain Heart Infusion Broth) (Sigma Aldrich, St. Loius, MO, USA) and incubated for 24 h at 37 °C. The medium was then diluted to an optical density of 0.1 at a wavelength of 600 nm (OD600 = 0.1). For bacterial adhesion, enough solution with a concentration equivalent to (OD600 = 0.1) to cover the surfaces (500 µL/sample) was introduced into the well of the culture plate of each sample and incubated at 37 °C for 1h. Sample incubation was carried out using an incubator oven MEMMERT BE500 (MEMMERT Gmbh, Scheabach, Germany). All assays were performed in static conditions without external stirring.

After this time, the samples were rinsed with PBS for 5 min twice, and the bacteria were fixed with a 2.5% glutaraldehyde solution in PBS (30 min in the refrigerator). The glutaraldehyde solution was then removed and the samples were rinsed with PBS 3 times for 5 min. For viability analysis by confocal microscopy, the LIVE / DEAD Backlight bacterial viability kit (Thermo Fisher, Spain) was used [74,75,76].

A solution was prepared with 1.5 μL of propidium in 1 mL of PBS. Using a micropipette, a drop of this solution (approximately 50 μL/sample) was deposited on the study surface and after incubation at room temperature in the dark for 15 min, the samples were rinsed three times with PBS for 5 min. The surfaces were then observed by laser scanning microscopy (CLSM). Three images per sample were taken at 630 magnification. A wavelength of 488 nm and 561 nm, respectively, was used to detect live and dead bacteria. This study has allowed us not only to analyze bacterial viability on each surface, but also to make an initial comparison of the number of bacteria present in each group of samples.

Prior to the observation of the samples by electron microscopy (SEM), the samples were dehydrated. For the dehydration process, 10 min washes were carried out with ethanol solutions of gradual concentrations of 30%, 50%, 70%, 80%, 90%, 95%, and 100%. They were then left to dry for 24 h at room temperature. As the surfaces are not very conductive, ion sputter Pt–Pd nano coating was conducted onto dehydrated and dried surface was deposited using Hitachi E1030 equipment (Hitachi High-Tech Europe GmbH, Krefeld, Germany) to allow properly SEM observation. Ten images of each sample were taken at 20,000 magnifications for bacterial quantification on each surface. Calculations were expressed in colony-forming units (CFU) expressed per surface for comparison between the different groups of samples.

All results were expressed as mean and standard error except for the bacterial adhesion test results, which were expressed as median and standard error.

#### 2.2.6. Statistical Analysis

Statistical analysis was performed using the comparative T.TEST (with the Excel software), that was carried out between the different groups at 95% of confidence, which means that for values of (*p* < 0.05), there are statistically significant differences.

#### 2.2.7. Ethical Approval

The carrying out of this investigation did not need the approval and supervision of an Ethics committee.

## 3. Results

### 3.1. Surface Characterization

The chemical analysis of the surface before and after the different chemical treatments does not modify the presence of contaminating elements that are at the level of non-detectable traces. The titanium composition is 99.9% which corresponds to a c.p.-Ti and there is only a surface oxygen increase of 9 to 16% for the case of 20% and 40% citric acid, respectively. No other elements are detectable at a sensitivity of 0.1% on the titanium surface.

#### 3.1.1. Roughness

**Figure 2:**
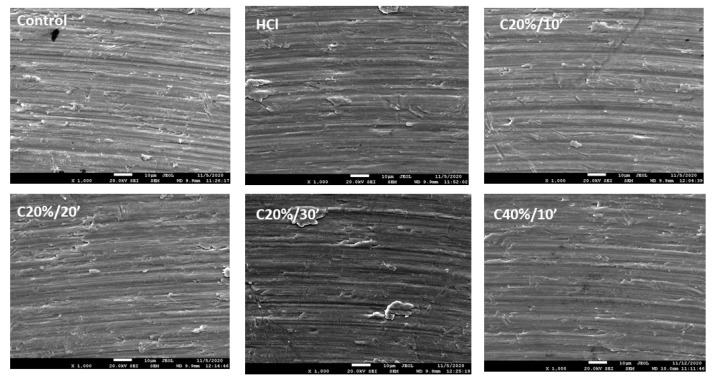
Surfaces of the cpTi treated with different passivation conditions.

Figure 2 shows the surfaces of the titanium discs after passivation treatments as observed by electron microscopy. No significant variations between the different treatments can be detected, showing the traces of machining. The observation would indicate that the machining scratches are lighter, probably due to the effect of the higher concentration of the acid.

The roughness measurements (Ra) reveal that the different passivation treatments with HCl and citric acid carried out on the Titanium discs do not affect the roughness, as no statistically significant differences (*p* < 0.05) were observed with respect to the Control group (Figure 2). The mean roughness values (Ra) of the samples evaluated in this study were between 0.12 μm (Control) and 0.16 μm (C20%30’), as can be observed in Table 2.

**Figure 3:**
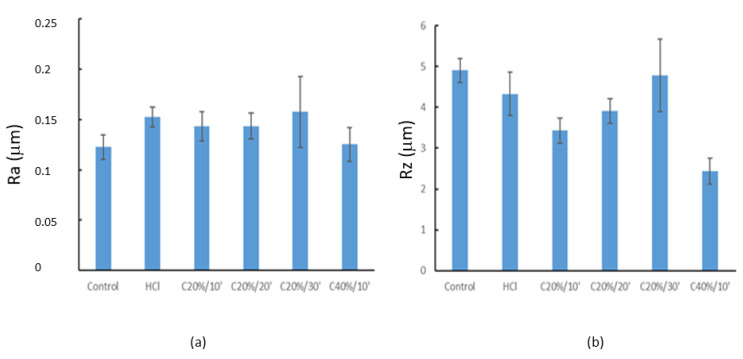
Roughness parameters of cp. Ti treated with different passivation conditions; (**a**) Ra, and (**b**) Rz.

The Rz measurements (Figure 3) have provided information on the mean peak-to-valley distance obtained as a function of the treatments. The groups showing statistically significant differences (*p* < 0.05) with respect to the Control group were C20%/10’, C20%/20’, and C40%/10’. C40%/10’ groups also showed statistically significant differences with respect to the other groups, with lower Rz values.

#### 3.1.2. Wettability

**Figure 4:**
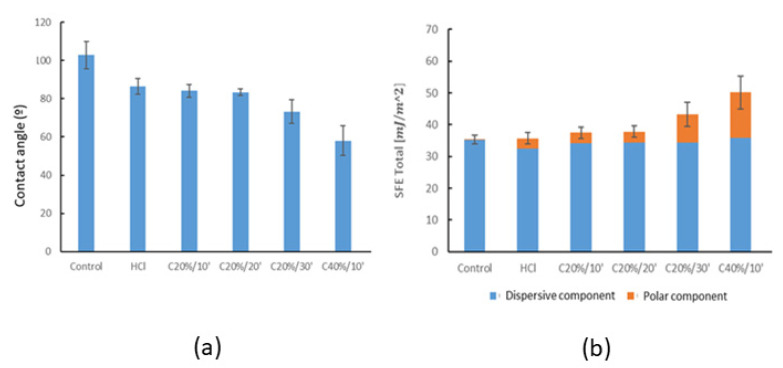
CA values (**a**) and SFE values (**b**) of cp. Ti treated with different passivation conditions.

The evaluation of wettability by determining the contact angle with the Sessile Drop technique has allowed the hydrophilic/hydrophobic character of the different surfaces studied to be determined. Firstly, it has been observed that the surface of the Control sample is hydrophobic since its contact angle exceeds 90° (Figure 4). Likewise, it can also be observed that all of the treatments evaluated have managed to increase the hydrophilicity of the surface with respect to the untreated Control sample. CA and SFE determined values are summarized in Table 3.

No statistically significant differences (*p* > 0.05) were observed in the contact angle between the HCl, Citric 10 mins, and Citric 20mins groups. The Citric 30mins group does show significant differences (*p* < 0.05) with respect to these three groups and the C40%/10’, which has lower angles (higher wettability).

The surface free energy (SFE) values obtained from the contact angles of water and di-iodomethane can be observed in Figure 4, in which dispersive and polar components of SFE are differentiated.

### 3.2. Corrosion Behaviour

The group of samples with the highest open-circuit corrosion potential (Eocp) values corresponded to the C20%/30’ as can be observed in Table 4. The detailed analysis of the results obtained for the groups passivated with citric acid showed an increase in the E_OCP_ value towards more electropositive (noble) values with increasing immersion time in citric acid at 20% concentration. However, the high dispersion of the results obtained prevented the identification of statistically significant differences in (Eocp) between the groups evaluated.

The only groups showing different values from the others are C20%/20’ and C20%/30’. The Eocp values of the Control and HCl groups are practically the same, which is surprising since HCl passivation is a treatment commonly used to improve the corrosion resistance of the material.

The electrochemical parameters obtained from the analysis of the potentiodynamic curves and their Tafel’s slopes are shown in Table 5. It should be noted that there are statistically significant differences in corrosion potential between the citric acid passivated titanium and the control. Also noteworthy are the statistically significant differences between the samples treated with 20% citric acid for 30 min and the rest of the samples studied.

This group shows better corrosion properties with a lower corrosion current density (icorr) and corrosion velocity (Vc), as well as a higher resistance to polarization (Rp). The HCl group has similar values for both icorr and corrosion rate, but its polarization resistance is not as good, indicating that it is more sensitive to small variations in potential.

**Figure 5:**
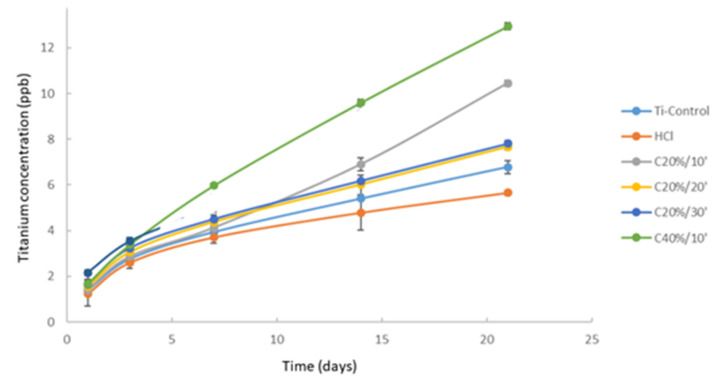
Ion release at different immersion times in Hank’s solution of different passivation treatments on c.p.-Ti.

Figure 5 shows the Ti ion release curves in the liquid medium used, Hank’s solution, as a function of incubation time expressed in days. The results show the cumulative Ti concentration in parts per billion (ppb) as a function of time.

The group of samples that showed the lowest release of Ti ions corresponded to the group of samples with HCl passivation treatment, with a total cumulative concentration after 21 days of incubation of 6.66 ppb. This treatment did not show statistically significant differences (*p* > 0.05) with respect to the Control group (6.78 ppb).

The statistical analysis of the results did show the presence of significant differences in the release of ions from the other groups of samples with respect to the control group. The C20%/10’ and C40%/10’ groups show a more significant increase in their released concentrations.

The C40%/10’ group released the most Ti ions after 21 days (12.94 ppb), which represents more than twice the value of Ti ions released by the HCl group.

### 3.3. Bacterial Adhesion

**Figure 6:**
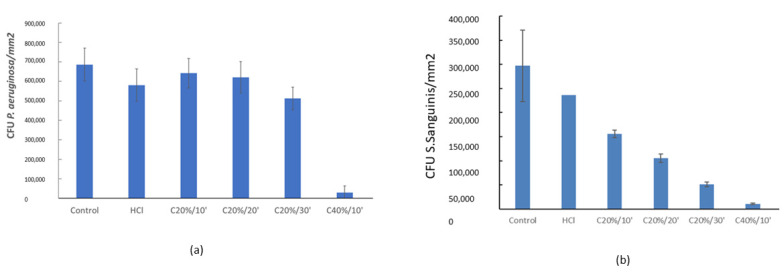
Analysis of P. aeruginosa (**a**) and S. sanguinis (**b**) adhesion for the different treatments.

Quantitative analyses of the bacterial adhesion assay performed with the Gram-negative strain *Pseudomonas aeruginosa* show that there are no significant differences (*p* > 0.05) in the number of bacteria adhered to the surface of the Control, HCl. and C20%/10’, C20%/10’ and C20%/10’ groups (Figure 6a).

**Figure 7:**
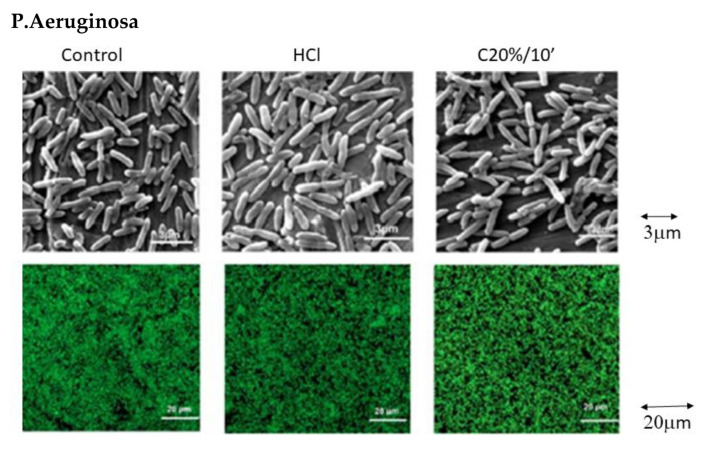

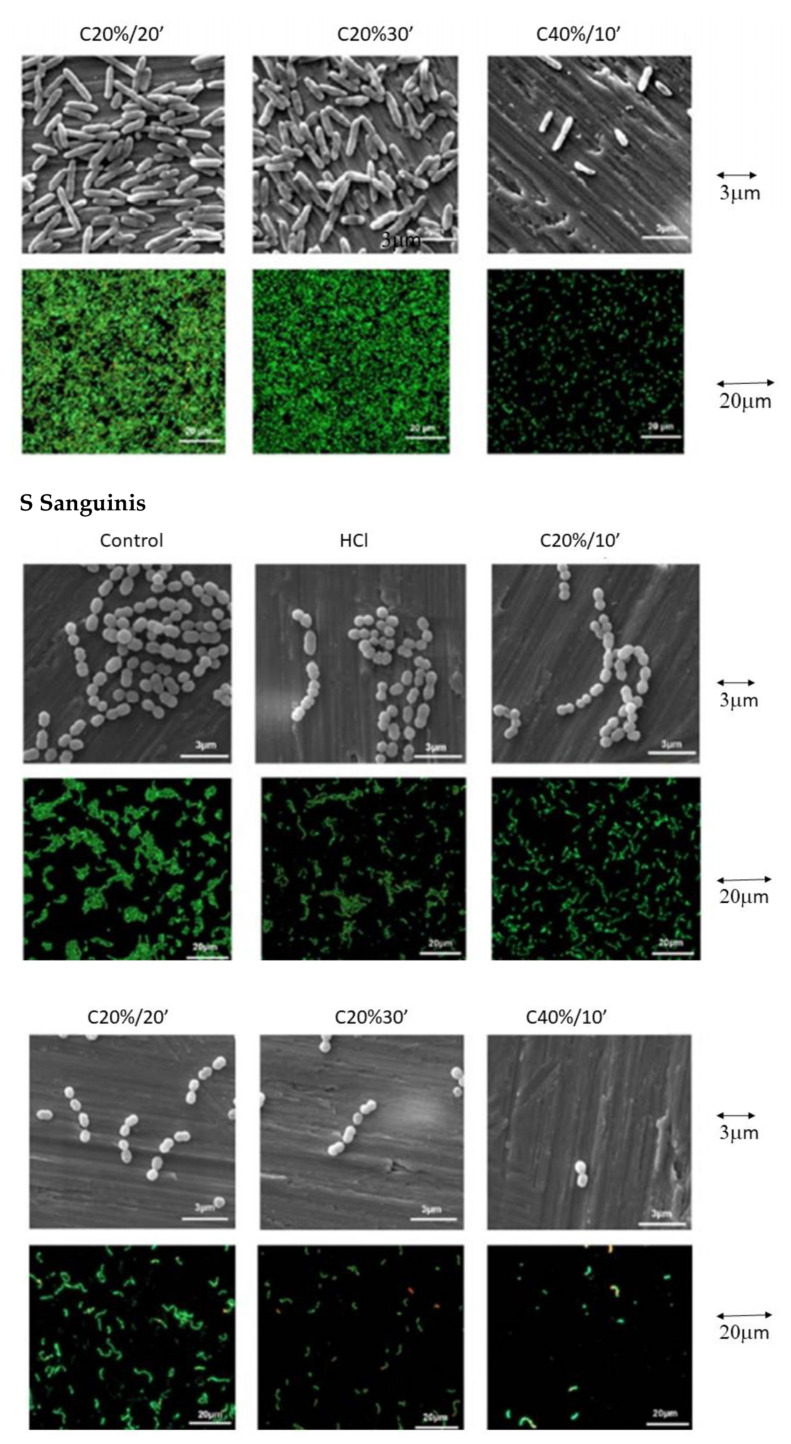
SEM and CLSM Microscope images of bacterial strains stained using Live/Dead^®^ BacLight bacterial viability kit which allows the assessment of the bacterial viability on each condition. Live = green and dead = dark red.

However, the number of attached bacteria decreases drastically for C40%/10’. Both SEM micrographs and images taken by confocal microscopy (‘Life and Death’) (Figure 7) clearly show this difference in bacterial adhesion.

Likewise, the analysis of the results obtained for the Gram-positive *Streptococcus sanguinis* strain showed a clear trend towards a reduction in bacterial adhesion with increasing exposure time and citric acid concentration (Figure 6b).

Statistically there are no significant differences (*p* > 0.05) between the Control, HCl and C20%/10’ groups. The samples treated with C40%/10’ show low bacterial adhesion (Figure 6) with no significant differences between them, but with large statistically significant differences (*p* < 0.05) with respect to the rest of the groups (Figure 7).

For both bacterial strains tested in this study, the C40%/10’ treatment showed lower bacterial adhesion than the other groups.

## 4. Discussion

The C40%10′ group has presented more uniform surfaces than the rest of the groups in the SEM micrographs (the machining marks are somewhat smoother), so from this point of view it can be understood that the Rz values obtained are lower. These results may suggest that in the treatment with 40% citric acid has etched the titanium to a certain extent or the oxide layer formed is thicker, reducing the differences between the peaks and valleys.

From the wettability results, all of the passivation treatments tested increased hydrophilicity. Consequently, it increases the interaction between the implant surface and the biological environment, favoring cellular activity and bacterial adhesion. In general, cell adhesion and proliferation increase on hydrophilic surfaces. In particular, fibroblasts are sensitive to variations in wettability; cell spreading increases the more hydrophilic the surface [77,78]. In the case of bacterial adhesion, this relationship is not so obvious as it depends on many factors, including the type of bacterial strain. This project has not taken cellular activity into account and has focused on the bacterial adhesion part, as this is an aspect of great relevance in the case of dental abutments.

In the specific case of citric acid passivation, the analysis of the results would allow a relationship between time/concentration and contact angle to be established. With the C20%/30’ group, the hydrophilic character of the sample increases with respect to the C20%/10’ and C20%/20’ groups, and it was therefore possible to deduce that the immersion time in citric acid after a certain time increases the wettability of the sample. The same trend was observed with respect to the concentration of citric acid, which was reflected in an increase in the wettability of the surface with increasing acid concentration. Likewise, the comparative analysis of results has allowed us to observe a greater influence of the citric acid concentration with respect to the immersion time on the surface wettability.

The analysis of the surface energy has allowed us to observe a certain relationship between the contact angle (CA) and surface energy (SFE) values. Low water contact angle values imply high surface energy levels. As they are related, both contact angle and surface energy values depend on the same parameters: surface chemistry of the substrate (determines to a greater extent polar and dispersive interactions), surface topography (crystallography, porosity and roughness), and fluid characteristics. These parameters determine the interaction between the implant and the biomolecules present in the physiological environment [57,79].

At the surface energy level (SFE), all surfaces have shown a dominance of the dispersive component over the polar component (Figure 5). As the surface energy (SFE) is equal to the sum of its dispersive and polar components, it has been observed that the differences between the total surface energy values are mainly due to the differences in the polar components, as the dispersive components are very similar for all of the groups. It is widely accepted in the literature that increasing the polar component of the surface energy of a material promotes initial adhesion and cell proliferation [80].

Corrosion resistance, both for open circuit and potentiodynamic samples, showed the best performance was C20/30’. These results showed no differences with HCl passivation. Samples with higher citric acid concentration give worse results as the layer produced is more porous due to the acid attack. The porosity allows areas susceptible to chemical attack. However, as we have seen, those with the highest citric acid concentration are the most bactericidal of all. The more acidic character prevents bacterial adhesion on the surface. Also, the high capacity for citrate formation makes the surface very reactive to bacterial adhesion.

The comparative analysis of the ion release curves showed a similar behavior in four of the sample groups evaluated (Control, HCl, C20 min, and C30 min), characterized by the presence of a first initial stage of strong ion release during the first three days of incubation, followed by a second stage of progressive stabilization of the ion release level between 3 and 21 days of incubation. However, the groups C10 20%, C10 40%, despite presenting an initial stage identical to the first four groups, did not show a clear stage of stabilization of the ion release level over time.

Different studies have reported that the blood concentration of Ti below which it is not considered toxic is 15.5 ppb [81,82]. In this study, the maximum concentration obtained was 12.94 ppb corresponding to the C40%/10’ group, not far below the toxicity value, so for the groups that do not show a clear stage of stabilization of the ion release level over time (C20%10′ and C40%10′) it would be interesting to perform future release studies with longer times to determine whether or not the release stabilizes over time.

The higher ion release shown by the C20%/10’ treatment compared to the Control, HCl, C20%/20’ and C20%/30’ groups could be related to the fact that these were the two groups with the highest corrosion rate and current density values. Corrosion phenomena are the main cause of the degradation of the passive layer and the subsequent release of ions into the medium.

In the groups passivated with citric acid, it can be observed that longer immersion times imply less bacterial adhesion. In the case of P. aeruginosa, the decrease in bacterial adhesion is not statistically significant among the groups passivated with citric acid 20%v, while for *S. Sanguinis* it is. The analysis of the results also showed that an increase in citric acid concentration causes a drastic decrease in bacterial adhesion. Thus, in view of the results, it could be stated that the concentration of citric acid has a greater influence than the immersion time on the behavior towards bacteria.

There is a possible explanation for the relationship between contact angle results and bacterial adhesion results. Some studies show that there is a relationship between surface hydrophobicity and bacterial adhesion [83]. Hydrophobic metal surfaces favor adhesion of hydrophobic bacteria. *S. sanguinis* are hydrophobic bacteria as are *P. aeruginosa*, so a decrease in bacterial adhesion could be correlated with an increase in surface hydrophilicity, as observed in our results [84] (Figure 6 and Figure 7).

The action of citric acid is related to its high concentration, which reduces the pH of the extracellular matrices. This acidification of the medium probably changes the membrane permeability of bacterial cells, changing the hydrogen gradient between intracellular and extracellular sites. Passivation with citric acid on cpTi surfaces yields a passivation layer with a thickness of about 6 nm in which Anathase and Rutile are found [56]. Improvements in corrosion resistance have been obtained for implants passivated until 40%, obtaining slightly higher corrosion potential values and a decrease in current and passivation intensities [54,55,56]. A possible explanation for these results lies in the way citric acid acts. It is possible that the citric acid acts first by degrading the natural oxide film and then interacts with the surface to form a TiH_2_ layer and subsequently re-forms a TiO_2_ layer [53,56,85].

In this research, however, a more extensive study should be carried out with other types of bacteria sensitive to periimplantitis and the behavior of the citric acid passivation layer with the biofilm should be studied. Further concentrations should be studied and the change in nanostructure created by treatment with high concentrations of citric acid should be determined. It seems that the layer could be porous and, therefore, the release of titanium ions into the medium would be higher.

## 5. Conclusions

Citric acid passivates result in a more hydrophilic surface and higher surface energy which makes them more biologically reactive. However, the roughness increases slightly but without statistically significant differences regarding control group. The citric acid concentration of 20% citric acid in a 30 min of immersion produces the best corrosion resistance. The best bactericidal behavior is for the 40% acid concentration on both Gram+ and Gram– strains with a high efficacy. However, this high concentration decreases the corrosion resistance and releases more titanium ions into the physiological environment. These aspects should be considered by clinicians for long-term performance. Citric acid treatment improves the properties of the passivation layer on titanium dental implants compared to conventional HCl treatments. However, at high citric acid concentrations, the increase of ions released into the medium in the long term must be taken into account.

## Figures and Tables

**Table 1:** Chemical composition of Hank’s solution.

Chemical Product	Composition (mM)
K_2_HPO_4_	0.44
KCl	5.4
CaCl_2_	1.3
Na_2_HPO_4_	0.25
NaCl	137
NaHCO_3_	4.2
MgSO_4_	1.0
C_6_H_12_O_6_	5.5

**Table 2:** Roughness parameters values of the titanium treated samples.

Parameter	Control	HCl	C20/10’	C20/20’	C20%/30’	C40%/10’
Ra	0.12 ± 0.01	0.15 ± 0.01	0.14 ± 0.01	0.14 ± 0.01	0.16 ± 0.03	0.12 ± 0.02
Rz	4.90 ± 0.30	4.33±0.53	3.43 ± 0.31	3.91 ± 0.31	4.78 ± 0.88	2.43 ± 0.31

**Table 3:** Values (mean ± standard deviation) of contact angle of water (WA) and diiodomethane (DIIO), and the estimated surface energy (SFE) with their polar (*γ^P^*) and dispersive (*γ^D^*) components, for each surface treatment.

Sample	CA (^o^)		SFE (mJ/m^2^)		
	WA	DIIO	*ϒ*	*ϒ* *^D^*	*ϒ* *^P^*
Control	102.77± 7.00	48.40 ± 2.32	35.28 ± 1.35	35.15 ± 1.28	0.12 ± 0.12
HCl	86.38 ± 4.12	53.34 ± 0.92	35.70 ± 1.60	32.39 ± 0.52	3.31 ± 1.28
C20%/10′	84.06 ± 3.26	50.22 ± 1.34	37.46 ± 1.27	34.14 ± 0.75	3.31 ± 1.05
C20%/20′	83.43 ± 1.89	49.88 ± 1.99	37.82 ± 1.20	34.26 ± 1.23	3.56 ± 0.61
C20%/30′	73.26 ± 6.28	52.72 ± 2.99	41.77 ± 2.82	34.27 ± 1.69	9.03 ± 2.07
C40%/10′	58.05 ± 7.67	47.02 ± 1.63	50.14 ± 3.87	35.91 ± 0.88	14.22 ± 4.29

**Table 4:** Open circuit potential for the different passivation treatments.

Parameter/Sample	Control	HCl	C20/10’	C20/20’	C20%/30’	C40%/10’
E_OCP (mV)_	−196 ± 1	−195 ± 11	−223 ± 0	−165 ± 0	−141 ± 22	−210 ± 13

**Table 5:** Electrochemical parameters obtained from potentiodynamic curves.

Sample/Parameter	Ecorr (mV)	Icorr (µA/cm^2^)	*Rp* (MΩ/cm^2^)	Vc (µm/year)
Control	−196 ± 14	0.027 ± 0.008	2.428 ± 0.390	0.233 ± 0.066
HCl	−536 ± 39	0.020 ± 0.005	2.479 ± 0.083	0.176 ± 0.048
C20/10’	−401 ± 42	0.031 ± 0.005	1.866 ± 0.010	0.268 ± 0.043
C20/20’	−471 ± 81	0.025 ± 0.001	2.797 ± 0.306	0.223 ± 0.001
C20%/30’	−470 ± 24	0.018 ± 0.002	3.566 ± 0.699	0.159 ± 0.020
C40%/10’	−429 ± 21	0.024 ± 0.008	2.845 ± 0.770	0.214 ± 0.071

## Data Availability

Not applicable.
